# Fasting for male fertility—a mixed methods study

**DOI:** 10.3389/fnut.2024.1529466

**Published:** 2025-01-16

**Authors:** Katharina T. May, Jocelyn Behling, Katharina Sochiera-Plegniere, Katharina Batschari, Christian S. Kessler, Andreas Michalsen, Farid I. Kandil, Sarah B. Blakeslee, Michael Jeitler, Wiebke Stritter, Daniela A. Koppold

**Affiliations:** ^1^Institute of Social Medicine, Epidemiology, and Health Economics, Charité - Universitätsmedizin Berlin, Corporate Member of Freie Universität Berlin and Humboldt-Universität zu Berlin, Berlin, Germany; ^2^Department of Internal Medicine and Nature-Based Therapies, Immanuel Hospital Berlin, Berlin, Germany; ^3^Charité Competence Center for Traditional and Integrative Medicine (CCCTIM), Charité – Universitätsmedizin Berlin, Corporate Member of Freie Universität Berlin, Humboldt-Universität zu Berlin and Berlin Institute of Health, Berlin, Germany

**Keywords:** fasting, healthy lifestyle, semen analysis, sperm motility, infertility, male

## Abstract

**Purpose:**

Approximately 10–20% of couples in Germany are unable to conceive. About 50% of this subfertility can be attributed to the male partner. Preclinical studies suggest that fasting could potentially influence central mechanisms of spermatogenesis. This study aimed at investigating feasibility and effects of a Fasting Mimicking Diet (FMD) in the context of male subfertility.

**Materials and methods:**

In this two-arm, randomized, controlled, exploratory mixed methods study men with impaired sperm quality were randomized into a fasting and a waiting-list control group. The fasting group followed an FMD (500 kcal/d for 5 days) thrice within 4 months, while the control group was instructed to maintain their lifestyle and diet. We assessed sperm quality according to WHO criteria (total and progressive sperm motility, concentration, total sperm count, ejaculation volume and sperm morphology) from baseline to 6 months later. Semi-structured interviews were conducted in a subgroup and evaluated by structured content analysis.

**Results:**

Recruitment proved difficult, with 18 out of only 22 recruited participants completing all visits. There were no marked group differences between fasters (*n* = 10, 36.9 ± 5.17 years) and controls (*n* = 8, 36.1 ± 2.8 years) regarding sperm parameters. Effect sizes suggest slight positive trends regarding between group changes in the ANCOVA for total sperm motility (eta^2^ = 0.030) progressive sperm motility (eta^2^ = 0.059), total sperm count (eta^2^ = 0.001), concentration (eta^2^ = 0.050), normal sperm morphology (eta^2^ = 0.019) and the percentage of round cells (eta^2^ = 0.462) in the fasting group and a general decrease of sperm quality in the control group. This decrease of sperm quality concerned all parameters but the ejaculation volume, which increased in the CG but decreased in the FG (eta^2^ = 0.254). The decline of sperm quality in the CG is not explicable by the study setting. We also saw positive trends concerning the intragroup changes (e.g., within group change for progressive sperm motility: d = 0.36), Qualitative analysis (10 interviews) showed FMD feasibility, and its compatibility with full-time work. Motivation toward a healthier lifestyle after the FMD and a feeling of self-empowerment concerning one’s fertility were reported.

**Conclusion:**

This limited exploratory study showed FMD feasibility but found no notable differences between groups regarding all parameters. Yet, we saw positive trends regarding the between and within group changes in favour of the fasting group. Possible beneficial effects of the FMD on sperm quality should be investigated in larger studies. Interview results suggest that fasting could be a useful supportive intervention in male subfertility regarding self-efficacy and positive lifestyle changes.

## Introduction

Approximately 10–20% of couples worldwide are unable to conceive children (1). Infertility can be diagnosed when intercourse without contraception does not lead to a pregnancy for over 1 year (2). In 2016, roughly 3% of live births in Germany resulted from artificial insemination (ART), with an average cost of about 37.000€ for a successful pregnancy (3).

In around 50% of cases, the couple’s infertility can be attributed to the male partner (1, 4, 5) Although this fact is largely known, interventional options predominantly address women (4).

Male subfertility can have various causes and manifests itself in compromised sperm quality, evaluated by WHO guidelines based on specific criteria such as total sperm count, sperm motility and ejaculation volume (6). Sperm quality is significantly affected by lifestyle and is subject to high fluctuations even in fertile men (7). Psychological stress disturbs hormonal balance and can lower luteinizing hormone and testosterone and thus impair sperm quality (8, 9). Often the unfulfilled desire for children creates severe mental pressure, so that stress can be both cause and consequence of subfertility (10).

Non-pharmaceutical therapeutic options recommended for enhancing sperm quality include acupuncture, exercise, lycopene, *ω*-3 fatty acids, Coenzyme Q10, zinc, vitamins, selenium, carnitine, or diets rich in these nutrients, which seem to be beneficial in addressing male infertility (11). Fasting interventions have not been studied in this context yet.

**Figure 1:**
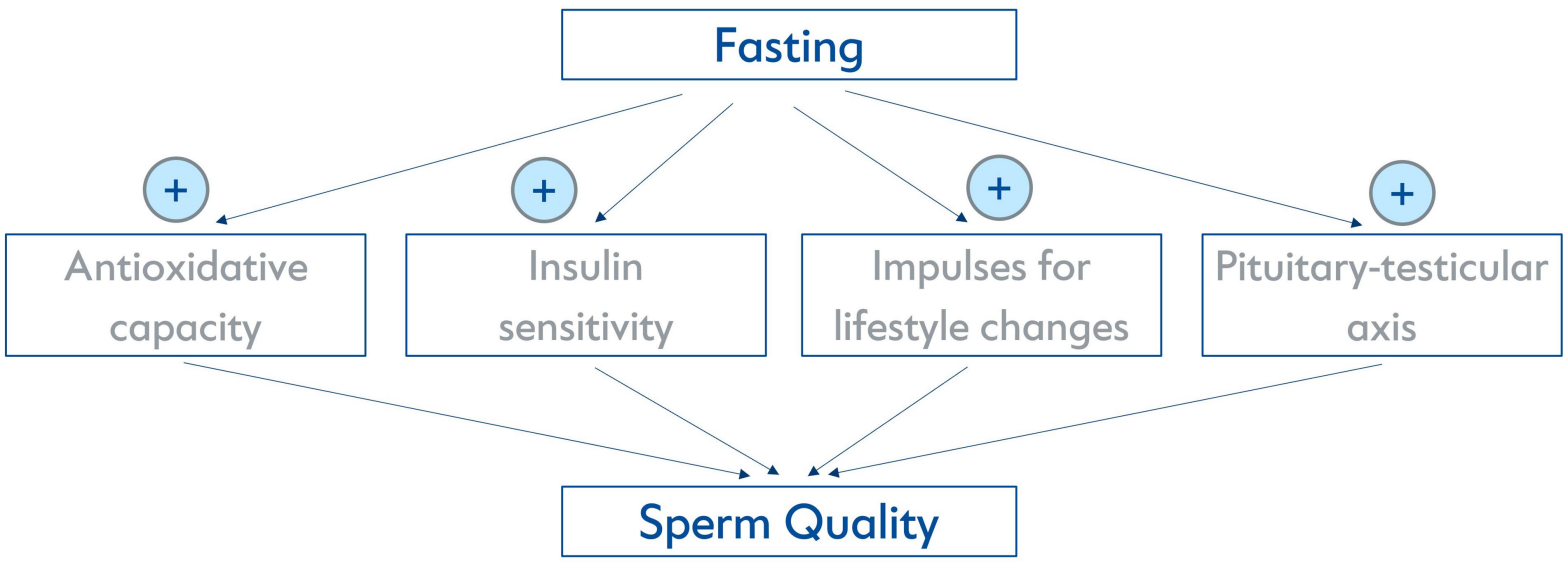
Possible biomolecular mechanisms on how fasting might affect sperm quality.

In general, medical fasting is increasingly being investigated experimentally and clinically. Prolonged fasting and the fasting-mimicking diet (FMD) is gaining popularity as a non-pharmacological method of prevention and therapy in Central Europe and the US (12–15). While there is an abundance of experimental studies that describe anti-inflammatory, beneficial cardiometabolic and cellular hormetic effects (16, 17), there have been no preclinical or clinical studies specifically examining the influence of fasting on sperm quality. However, from a physiological point of view, several mechanisms activated by fasting could possibly influence it. Fasting has been proven to decrease oxidative stress and chronic inflammation (16). A (pre-) diabetic metabolic state has a negative impact on sperm quality (18) and fasting has been found to potentially increase insulin sensitivity (19). Furthermore, fasting has been proven to be a suitable impulse for lifestyle changes (20), which can be essential for improving sperm quality as described above. It is also known that spermatogenesis relies directly on testosterone levels (21) and potential positive influences of two days of food deprivation on the pituitary-testicular axis have formerly been described in a very small sample of obese and non-obese men (22). An overview of possible biomolecular mechanisms is illustrated in Figure 1.

This study explores fasting as a possible non-pharmacological approach addressing male subfertility. We used a mixed methods design to assess feasibility and effects of three FMD cycles for men with subfertility. To the best of our knowledge, it is the first clinical trial to investigate the effects of an FMD on sperm quality.

## Materials and methods

In this two-arm, randomized, controlled, exploratory mixed methods study, men with impaired sperm quality were randomized into a fasting group and a waiting-list control group. It aimed to investigate the feasibility of repetitive cycles of an FMD and its effects in a male subfertile population. It is part of a larger project investigating the effects of fasting on both male and female fertility conducted at the Charité-Universitätsmedizin Berlin Research group on Internal and Integrative Medicine. It followed the criteria of the Declaration of Helsinki as well as Good Clinical Practice guidelines, was approved by the institutional review board of Charité-Universitätsmedizin Berlin in December 2021 (ID: EA4/230/20), registered at clinicaltrials.gov and checked by the Mixed methods appraisal tool.

### Study setting

**Figure 2:**
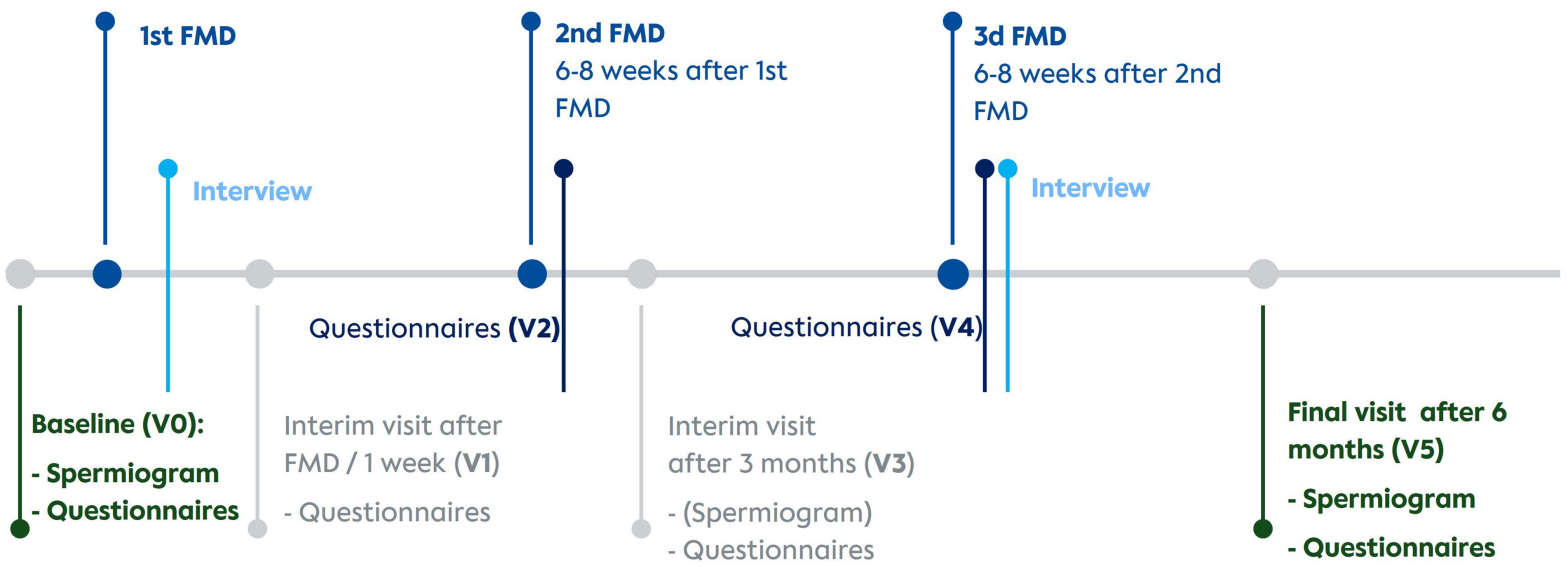
Timeline of interventions, visits and interviews. V0, Baseline visit; V1, Visit 1; V2, Visit 2; V3, Visit 3; V4, Visit 4; V5, Visit 5; FMD, Fasting mimicking diet.

Patients were referred by four cooperating fertility centres in Berlin. Eligible participants were assigned either to a fasting group (FG) or a control group (CG) in parallel assignment. Four visits (V0 = baseline, V1&V3 = interim visits, V5 = final visit) included questionnaires, spermiograms and a short general visit about health status and possible adverse events. Additionally, online questionnaires sent via email were to be answered by participants after the second (V2) and the third FMD cycle (V4). All visits were conducted online. The timeline of visits is presented in Figure 2.

**Figure 3:**
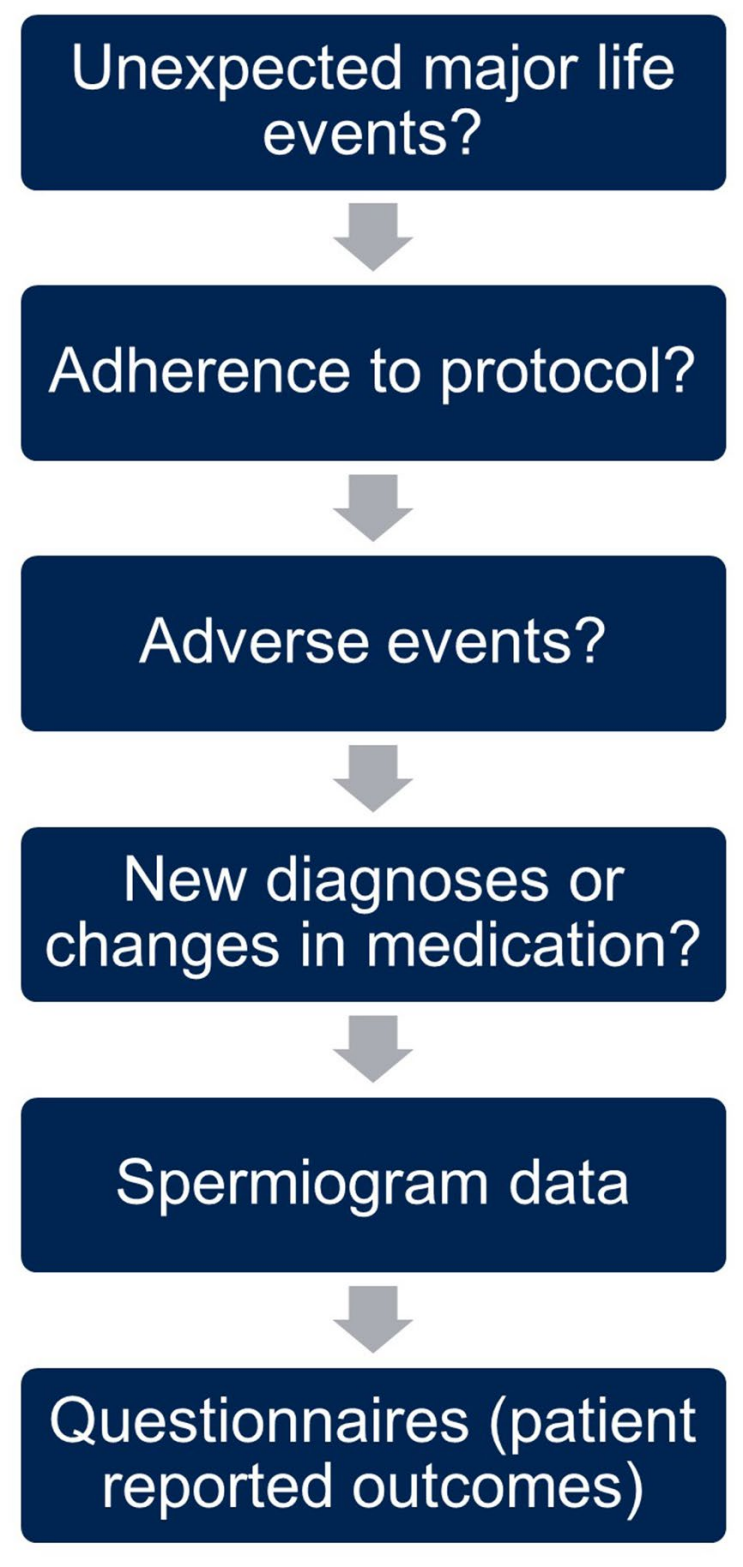
Content and structure of online visits.

The content and structure of the online visits is outlined in Figure 3. We began every visit with an open question encouraging participants to share experiences since the last visit, specifically major life events affecting the study, e.g., separation from the partner. This was followed by a short inquiry on the adherence to the assigned group protocol and possible adverse events. Further, we asked about new diagnoses or changes in medication as well as the data from the latest spermiogram. At the end of the visit, participants filled in questionnaires on wellbeing and their current lifestyle to capture changes in behavior.

The FG followed an FMD for 5 days every 6–8 weeks for a total of three times within 4 months. A company that developed standardized organic plant-based fasting-mimicking meals with a total daily energy intake between 500 and 630 kcal (for 5 days) provided FMD boxes and their mobile application for free to all participants (23). Through this mobile application participants were provided with both motivational and informational videos about their FMD meals as well as about fasting in general. Participants in the control group (=waiting-list) were instructed to maintain their usual diet during the duration of the study and not to change anything regarding their lifestyle. As an incentive, after completion of the study, they got the opportunity to fast under medical supervision.

Men aged between 18 and 60 years who (i) had not been able to have a child for at least 1 year, (ii) had impaired sperm quality proven in a spermiogram conducted according to WHO-5 criteria and (iii) had a BMI between 20 and 35 kg/m^2^ were included in the study. Interested men with organic damage of reproductive organs, a medical history of eating disorders, or serious internal or mental diseases were excluded.

Eligible participants were randomly assigned to either the fasting group (FG) or the waiting-list control group (CG) in a 1:1 ratio during the baseline visit. Randomization was carried out using a computer-generated randomization list on the database platform RedCap (Version 13.7.31) (24). This list was generated by and only accessible to the statistician. Due to the nature of the intervention, both participants and research staff were aware of the assigned intervention. Blinding was implemented for the staff conducting the assessment of spermiograms and statistical analysis.

Main quantitative outcomes were parameters for assessing sperm quality according to WHO-guidelines 2010 (6), as the study commenced before the publication of the new WHO manual in 2021 (25). The WHO manuals have been designed to unify assessment of sperm quality, such as progressive sperm motility, ejaculation volume, total sperm count, concentration, normal morphology and round cells.

Participants collected spermiograms during their routine fertility care in their respective fertility centres. Thus, timing was subject to practicability in the fertility centres.

The interview guideline for the qualitative part of the study (Figure 2) was developed according to Helfferich (26) and drew upon our previous experience with other fasting studies as well as the previously started quantitative part of this study. It addressed fasting experience, support during the study, motivation and the participants’ experience with their desire to have a child. At the outset of the study, all participants gave written informed consent for individual interviews and the interviews were conducted at various time points throughout the study. Interviewees were selected according to willingness and sufficient German skills. Based on availability, select participants were chosen to be successively interviewed twice to assess whether the repetition of the FMD had any impact on their reported experiences. To ensure practicality for participants, the interviews were conducted on the online platform Samedi in German, facilitated by a team comprising two medical students (JB and KTM). They were recorded and transcribed verbatim in accordance with content-semantic transcription guidelines (27). Transcriptions were controlled and anonymized by KTM.

### Data analysis

#### Semen analysis

The sperm samples were examined in the andrological laboratories of the treating urological practices or their respective fertility centres. The sperm samples were obtained by masturbation after 2–6 days of sexual abstinence and liquefied at room temperature. The parameters liquefaction time, ejaculate volume, pH, total sperm count, progressive and total motility, sperm morphology and proportion of round cells in the ejaculate were assessed according to the WHO guidelines of 2010 (6). Despite strictly standardized procedures, it can be assumed that the examination technique varied slightly in each laboratory. It was taken care to ensure that each participant performed their spermiograms in the same laboratory during the study, so that an individual change in the results would not be due to the change between laboratories.

### Statistical analysis

As fasting in this medical context has not been investigated so far, this study aimed to explore the various endpoints and their effects using a target sample size of 40 participants, i.e., 20 in each group. To this end and to allow a drop out-rate of 5%, 42 participants were to be recruited. All data were collected using a custom defined RedCap database (24). For the baseline characteristics, group differences in age, BMI, and sperm quality at baseline were assessed.

Group differences in the endpoint parameters were statistically assessed employing an ANCOVA between the values at V5 (post-test) with V0 (baseline/pre-test) values as covariates. Within-group changes between V0 and V5 were evaluated *post-hoc* for the experimental and the control group separately using paired *t*-tests. All analyses were conducted using scripts written in Python (3.12) and its statistical tools scipy (1.13.1) and statsmodels (version 0.14.2). As usual in explorative studies, assessments focused on effect sizes (eta-squared for the ANCOVA, and Cohen’s d for the *t*-test) rather than *p*-values. Effect sizes tend to have a higher informative value in this context than *p*-values. Thus, in the results section, we will focus on effect sizes.

### Qualitative analysis

The transcripts were analysed in the research software MAXQDA Plus (28). Initially, primary categories were established through deductive reasoning (by KTM, JB, WS, DAK) drawing upon the experience with the quantitative part of this as well as other fasting studies. In a subsequent step, subcategories emerged through inductive reasoning. To ensure plausibility and comprehensibility in category framing and results (29), all findings were regularly discussed within a qualitative research group.

## Results

A total of 22 participants were enrolled between May 2021 and September 2023 and evenly randomized into the two groups. They followed the study between May 2021 and September 2023. Due to severe challenges in recruiting participants during the COVID-19 pandemic, we expanded the study setting from a monocentre design to a multicentre approach and still had to stop recruitment after 2.5 years before reaching the planned number of participants. We had to adapt our originally fixed time points for spermiograms to the course of the routine fertility care, the extremes being one case with a 5-month-old baseline spermiogram and one final spermiogram being collected 2 months after the last visit. 45% of the participants had their spermiograms evaluated in the fertility centre “Praxis für Fertilität,” Berlin, Germany.

**Figure 4:**
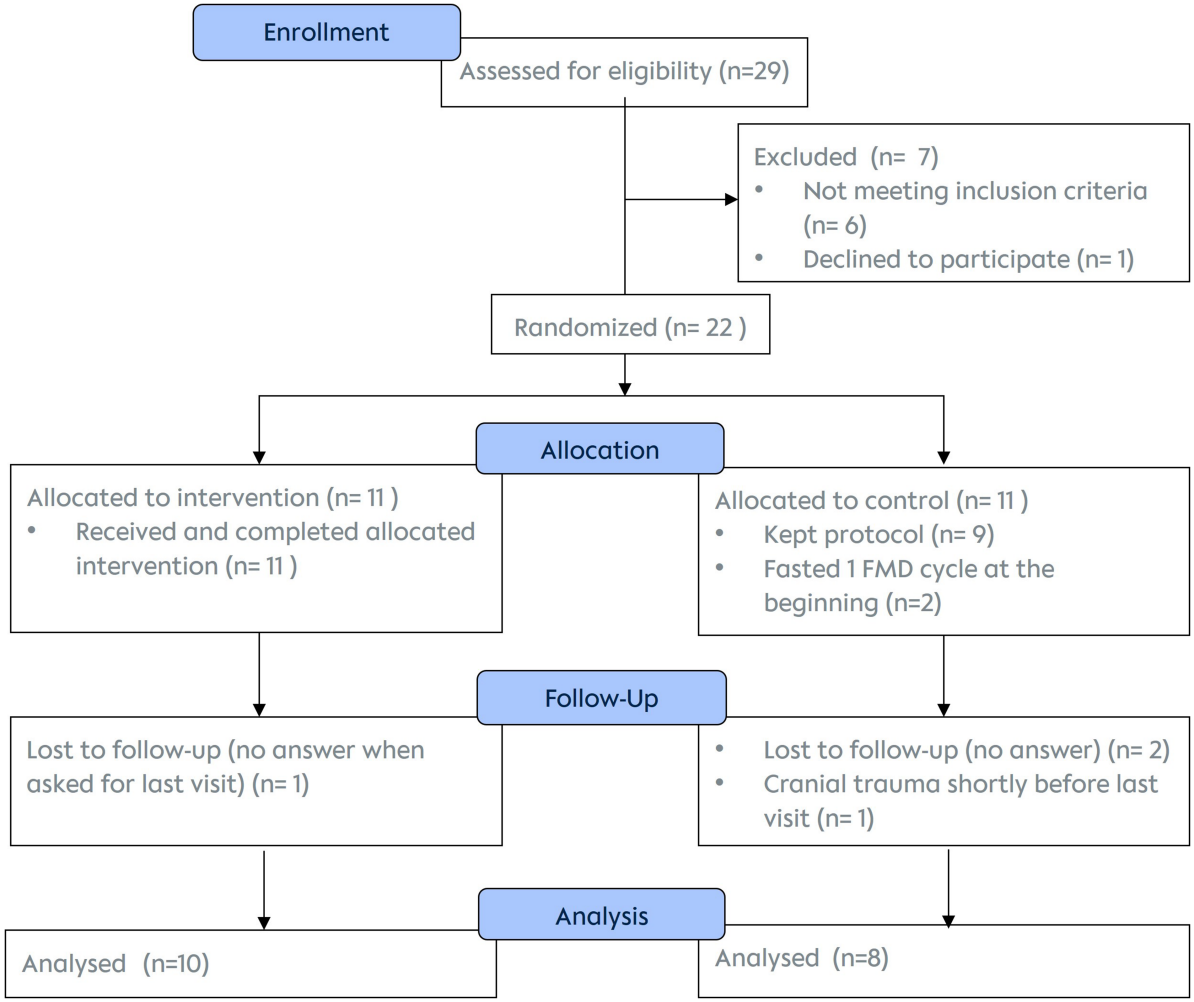
Consort flow diagram of participants.

Of the 22 enrolled men, data of 18 were included in the final analysis. While all 11 participants of the FG successfully completed all three cycles of dietary interventions, only 10 (and 8 controls) completed the 6-month follow-up by attending all visits, filling in questionnaires and providing spermiogram analyses (Figure 4).

All participants completing the last visit were included in the statistical data analysis. The two participants who did one fasting cycle at the beginning of the course of the study despite having been randomized into control group, were included in the data of the control group (intention to treat analysis).

### Baseline parameters

Baseline evaluation is reported in Table 1. Τhere were distinct differences concerning all parameters between the FG and the CG, possibly due to the small sample size. Sperm quality was overall lower in the FG than in the CG at baseline, with impaired values below WHO-references concerning total sperm motility (reference value: >42%) and progressive motility (reference value: >30%) group as well as normal morphology (reference value: >4%) (6).

**Table 1:** Baseline parameters.

Parameter	FG (*n* = 10)	CG (*n* = 8)	Reference value
Age (in years, SD)	36.9 ± 5.17	36.1 ± 2.8	18–60
BMI (in kg/m^2^, SD)	28.3 ± 4.57	26.5 ± 2.23	20–35
Total sperm motility (in %, SD)	30 ± 24.77	48.6 ± 24.07	≥42.0
Progressive sperm motility (in %, SD)	16.2 ± 12.42	34.7 ± 25.83	≥30.0
Total sperm count (in mio., SD)	53.0 ± 52.24	134.8 ± 122.82	≥39.0
Concentration (in mio/ml, SD)	24.8 ± 27,43	29.8 ± 28.66	≥16.0
Ejaculation volume (in ml, SD)	2.8 ± 1.12	4.4 ± 1.1	≥1.4
Normal morphology (in %, SD)	1.6 ± 0.88	2.2 ± 1.46	≥4%
Round cells (in mio/ml, SD)	1.0 ± 0.99	1.5 ± 1.38	≤1.00

SD, standard deviation; FG, fasting group; CG, control group, n, number.

### Quantitative outcomes

**Figure 5:**
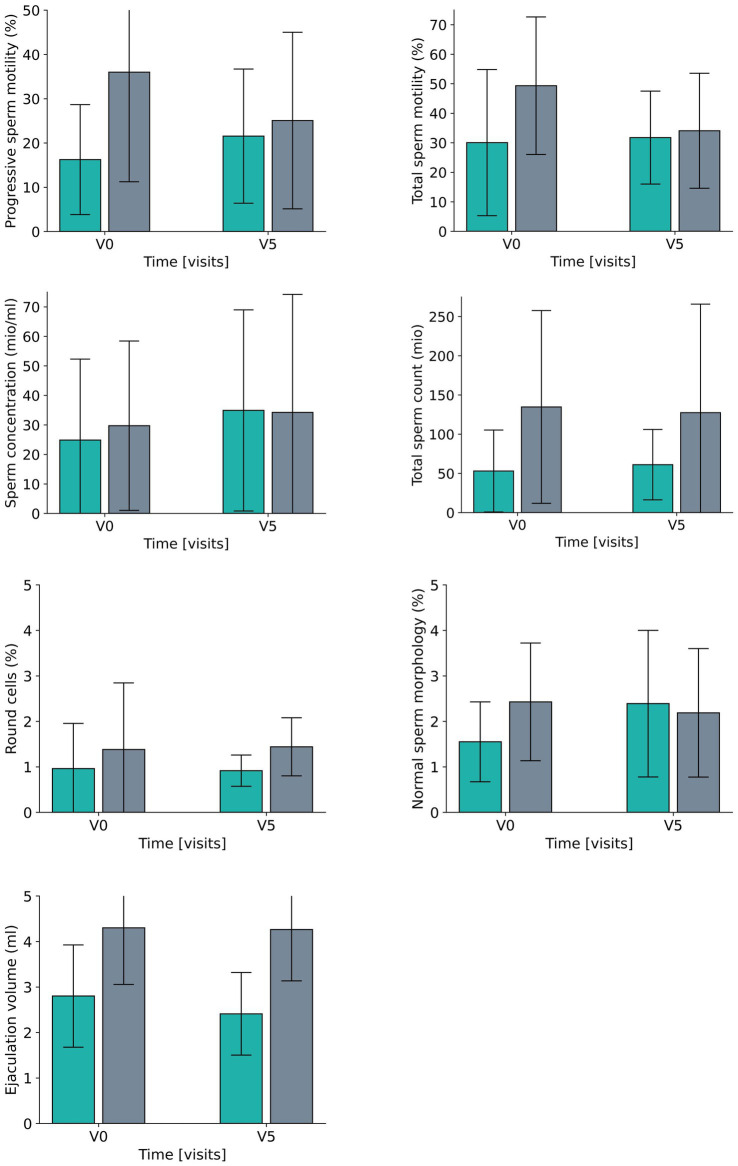
Development for all laboratory parameters between V0 (baseline visit) and V5 (after 6 months) for the fasting group (green) and the control group (grey).

As this was an exploratory study of small sample size, the analysis focused on the effect sizes for the results of both groups, the comparison between the two groups at V5 (Table 2) and the *post-hoc* analysis of the intragroup changes (Table 3 and Figure 5).

**Table 2:** Between group differences in all evaluated WHO-5 spermiogram parameters after 6 months.

Parameter (unit)	Group	*n*	V0		V5		Between-group ANCOVA			
			M	SD	M	SD	*F*	*p*	eta^2^	(d)
Total sperm motility (%)	FG	10	30.0	24.77	31.8	15.73	0.46	0.510	0.030	0.35 (small)
	CG	8	49.3	23.3	34.1	19.47				
Progressive sperm motility (%)	FG	10	16.2	12.42	21.6	15.15	0.94	0.349	0.059	0.50 (moderate)
	CG	8	36.0	24.76	25.1	19.93				
Total sperm count (mio)	FG	10	53.0	52.24	61.1	44.87	0.01	0.920	0.001	0.06 (−)
	CG	8	134.7	122.93	127.5	138.3				
Concentration (mg/dl)	FG	10	24.8	27.43	34.9	34.06	0.79	0.387	0.050	0.46 (small to moderate)
	CG	8	29.7	28.69	34.2	39.96				
Ejaculation volume (ml)	FG	10	2.8	1.12	2.4	0.91	5.10	0.039	0.254	1.15 (large)
	CG	8	4.3	1.24	4.3	1.13				
Normal morphology (%)	FG	9	1.6	0.88	2.4	1.61	0.25	0.623	0.019	0.28 (small)
	CG	7	2.4	1.29	2.2	1.41				
Round cells (%)	FG	5	1.0	0.99	0.9	0.34	5.15	0.064	0.462	1.85 (large)
	CG	4	1.4	1.46	1.4	0.64				

FG, fasting group; CG, control group; n, number; M, mean; SD, standard deviation; F, *F*-value; p, *p*-value; eta^2^, partial eta-squared effect size; (d), approximate estimation for Cohen’s d, recalculated from eta^2^ value, and its assessment following Cohen (1988).

**Table 3:** Intraindividual changes in sperm quality.

Parameter (unit)	Group	*n*	V0		V5		Difference V5–V0		Within-group *t*-test		
			M	SD	M	SD	M	SD	*T*	*p*	d
Total motility (%)	FG	10	30.0	24.77	31.8	15.73	1.7	21.84	0.23	0.821	0.08
	CG	8	49.3	23.3	34.1	19.47	−15.2	18.51	2.18	0.066	0.66 (M)
Progressive motility (%)	FG	10	16.2	12.42	21.6	15.15	5.3	15.23	1.04	0.324	0.36 (S)
	CG	8	36.0	24.76	25.1	19.93	−10.9	16.75	1.73	0.128	0.46 (S)
Total sperm count (mio)	FG	10	53.0	52.24	61.1	44.87	8.1	45.95	0.53	0.611	0.16
	CG	8	134.7	122.93	127.5	138.3	−7.2	96.18	0.2	0.848	0.05
Concentration (mg/dl)	FG	10	24.8	27.43	34.9	34.06	10.0	14.71	2.05	0.071	0.31 (S)
	CG	8	29.7	28.69	34.2	39.96	4.5	16.21	0.73	0.487	0.12
Ejaculation volume (ml)	FG	10	2.8	1.12	2.4	0.91	−0.4	0.75	1.55	0.155	0.36 (S)
	CG	8	4.3	1.24	4.3	1.13	0.0	1.45	0.07	0.947	0.03
Normal morphology (%)	FG	9	1.6	0.88	2.4	1.61	0.8	1.87	1.17	0.274	0.56 (M)
	CG	7	2.4	1.29	2.2	1.41	−0.4	1.76	0.6	0.573	0.29 (S)
Round cells (%)	FG	5	1.0	0.99	0.9	0.34	−0.4	0.80	1.1	0.331	0.51 (M)
	CG	4	1.4	1.46	1.4	0.64	0.8	0.69	2.08	0.129	1.02 (L)

FG, fasting group; CG, control group; n, number; M, Mean; SD, standard deviation; T, T-statistic of the paired-group *t*-test comparison; d, Cohen’s d; W, W-statistic of the Wilcoxon-test; p, *p*-value; CLES, common language effect size.

Data reveal a moderate difference (effect size of eta^2^ = 0.059/d = 0.50) between the two groups for the percentage of progressive sperm motility, which results from a smaller increase in the fasting group and a moderate decrease in the control group. The effects for the percentage of total sperm motility were a bit lower (eta^2^ = 0.030,/d = 0.35, small effect) and consisted of a moderate deterioration in the CG as opposed to a constant level for FG. In both groups the total sperm counts did not reveal any relevant effect between or within groups. Ejaculation volume evolved differently in the two groups (large effect size of eta = 0.254/d = 1.15) with a rather constant volume in the CG and a reduction the FG, while concentration changed accordingly over time (eta^2^ = 0.050/d = 0.46, small to moderate effects size) (Table 2; Figure 5).

For round cells, which are regarded as a general marker for inflammation or impaired spermatogenesis, there was a large effect (eta^2^ = 0.462, corresponding to a d = 1.85) for the difference between the groups, with a moderate reduction in the FG and a large increase in CG over time, while a complementary tendency was revealed for the percentage of cells with normal morphology – the effect size of the group differences was small and comprised a moderate-sized increase in the FG and a small-sized decrease in the CG.

#### Questionnaires

In the questionnaires, no associations were found between mood and general wellbeing and sperm quality in the unpaired *t*-test (Table 4). These results suggest that mood did not affect other results. BMI slightly decreased in both groups.

**Table 4:** Between Group differences in questionnaires.

Parameter	Group	*n*	V0		V6		Difference		ANCOVA		eta^2^	(d)
			M	SD	M	SD	M	SD	*F*	*p*		
ASTS	FG	10	61.6	7.55	60.5	8.4	−1.1	10.88	0.02	0.8893	0.0013	0.0
ASTS	CG	8	57.4	6.78	59.0	6.05	1.6	4.44				
WHO-5	FG	10	13.9	3.98	16.5	2.46	2.6	4.0	1.02	0.3276	0.0639	0.07
WHO-5	CG	8	13.8	4.92	14.6	5.32	0.9	4.91				
MAAS	FG	10	4.1	0.65	4.0	0.79	−0.1	0.46	0.04	0.8483	0.0025	0.0
MAAS	CG	8	4.5	1.02	4.4	1.01	−0,1	0.67				
ASKU	FG	10	4.2	0.56	4.1	0.47	−0.0	0.51	0.15	0.7073	0.0097	0.01
ASKU	CG	8	4.4	0.59	4.3	0.48	−0.1	0.61				
GRAW	FG	10	54.8	13.32	45.7	6.8	−9.0	13.53	0.43	0.52	0.0281	0.03
GRAW	CG	8	51.2	16.24	47.6	24.14	−3.6	13.0				
PSS	FG	10	16.9	2.6	17.9	3.9	1.0	4.34	1.55	0.2323	0.0936	0.1
PSS	CG	8	18.2	5.6	16.6	6.3	−1.6	2.64				
HADS Depression	FG	10	6.0	1.94	7.8	2.49	1.8	2.71	1.07	0.3169	0.0667	0.07
HADS Depression	CG	8	7.1	2.59	7.2	3.41	0,1	2.09				
HADS Anxiety	FG	10	6.2	1.87	6.9	1.45	0.7	2.33	0.04	0.842	0.0027	0.0
HADS Anxiety	CG	8	6.8	3.69	7.5	4,21	0.8	2.38				
BMI	FG	10	28.3	4.82	28.1	4.72	−0.2	0.75	0.27	0.6128	0.0175	0.02
BMI	CG	8	26.5	2.38	26.1	2.72	−0.4	0.8				

FG, fasting group; CG, control group; n, number; M, mean; SD, standard deviation; F, *F*-value; p, *p*-value; Eta-2, Pearson-correlation-coefficient; d, Cohen’s d; U, *U*-value of the Mann–Whitney-*U*-test; CLES, common language effect size.

### Qualitative results

Ten semi-structured interviews were conducted during the intervention period in an iterative process. Four interviews were carried out after the first FMD cycle, one after the second and five after the third. Three participants participated in two interviews (one after the initial FMD cycle and another following the last fasting intervention) to assess effects resulting from repetition. The main results along with pertinent quotes are presented in Table 5.

**Table 5:** Main qualitative findings accompanied by pertinent quotes.

Main category	Main finding	Pertinent quote
Feasibility	Every participant in the fasting group successfully completed all three FMD cycles. The interviews reflected that implementing the FMD during work is feasible. However, several participants emphasized the importance of forward planning to ensure a smoother experience. Specifically, scheduling the first fasting day on a Friday was deemed beneficial since it allowed the more challenging second and third fasting days to coincide with a weekend. Furthermore, most participants reported a habituation effect, meaning that the third FMD cycle was perceived as more manageable compared to the first cycle. Nevertheless, some participants disliked the repetition of the meals in the Salufast boxes. They wished for more variety in the meals especially in the third fasting cycle.	*“So, I already felt like a professional. As if I knew what I was doing. And then I also already knew: now it’ll be the time and then there might come a time of feeling less energetic – that’s nothing abnormal. This made me feel more confident during the whole process.” (B6 (2), after last FMD cycle, transcript, item 63)*
Sustainable lifestyle changes	Following the initial fasting cycle, participants expressed an intention to adopt healthier and more balanced eating habits. After completing the final fasting intervention, several participants indicated that they had increased their water intake and consumed smaller food portions due to a reduced stomach size and their recognition that they did not require as much food. Nearly all participants stated their intention to continue fasting even after study conclusion, either by maintaining an FMD or engaging in prolonged fasting.	*“I simply realized that a person does not need so much, just simply much less. […] Because I do not need this sugar crap either. Excuse me for saying this so radically, but I do not have any desire for it. Just cut out junk food. Radically leave out junk food.” (Transcript B17 (2), item 103)*
Interaction between fasting and the wish for a child	Given the limited therapeutic options for male subfertility, many men reported feeling powerless and believing there is little within their control to do to enhance their fertility before our study. Thus, interviews indicating that fasting provoked a sense of self-efficacy regarding fertility and motivated participants to make positive changes in their lifestyle to improve it are encouraging. One patient described fasting as such a profound experience that it prompted him to reflect on his own perception of his subfertility and his own ability to take action. On the other hand, all participants fasting for the first time reported that the unfulfilled wish for a child was the decisive reason for fasting. Their wish to have a child and their hope for the interventions´ effect also gave them the motivation to continue during tougher periods. Those who had previous fasting experience also described other reasons for their fasting such as general health promotion.	*But what [the fasting] has also shown me once again is that I myself am actually much more in control. So, I decide how I eat, I decide whether I exercise or not. (B08 transcript, item 438)* *[Fasting has shown me] that I can perhaps change something myself [in regard to subfertility] through my own effort. (B11 transcript, item 165–166)* *If I’m not really active now, nothing will change [in regard to subfertility…] The thing [fasting] has definitely done is to really actively look at [my perception of my own subfertility] again. (B08 transcript, item 415–423)* *“It wasn’t difficult for me. I knew what I was doing it for and I’m very ambitious about such things. And if you know what you are doing it for, then it’s not difficult to stick to it.* “*(B11 transcript, item 92)* *“Then I got myself out of [a physically and mentally challenging moment during fasting] by telling myself what I was doing it for. “(B08 transcript, item 28)*

## Discussion

In this mixed methods study we investigated the effects and feasibility of three FMD cycles on male subfertility. Total and progressive sperm motility as well as total sperm count, concentration and normal morphology showed positive trends in the fasting group, while ejaculation volume decreased. The intervention was feasible and safe in our studied population. Especially with regards to self-efficacy and motivation for positive lifestyle changes, fasting might be a promising supportive intervention in male subfertility.

This study has several limitations. Certainly, one limitation is the relatively small number of participants due to severe recruitment problems. We conducted the study during the COVID-19 pandemic, which complicated recruiting. Possible additional reasons could have been a hesitation on the part of the fertility clinics to endorse a fasting intervention and difficulties to implement it during fertility treatment. To get more insight into these possible background problems the authors of this study have carried out a qualitative research initiative at the fertility clinics, the results of which will be published separately. Unfortunately, we could not offer our participants financial incentives, resulting in a higher dropout rate in the control group. It also seems evident, that especially men with the motivation to undergo fasting, an intervention that might be perceived as drastic, were less motivated to complete all visits and spermiograms when randomized to the control group. The heterogeneity of the spermiograms being conducted at different fertility centers and sometimes also different time points due to restrictions of routine fertility treatments is another limitation, as is the changeability of the spermiogram itself (1, 30). Also, at baseline the groups showed differences in most of the parameters, so that they were not genuinely comparable. High effect sizes between the controls and fasters seem to be caused by an unexpected deterioration of the CG spermiograms during the course of the study. These changes in the control group are not explicable by the study setting and might be due to other changes in lifestyle or underlying fluctuations. This phenomenon was also seen in another study on sperm quality and were not further explained there either (31). Although we tried to track other lifestyle changes by questionnaires and also by addressing the subject personally in the online visits, it is not possible to exclude other lifestyle changes as a confounder in this study. Additionally, the FMD boxes used in this study were kindly made available by the SALUFAST company, in which one of our co-authors is a shareholder. It is obvious that all these limitations need to be considered when interpreting the results. Thus, this study should be regarded as a first stepstone, creating the base for future studies in this field, which then might provide more reliable and robust data on the effects of fasting on sperm quality.

To our knowledge, this study is the first to investigate the effects of fasting on subfertility. The completion rate of the fasting intervention by all participants tangibly shows the practicability of the fasting approach used. The mixed methods approach allowed for a wider exploration of the feasibility of the intervention and the subjective effects on the participants. The FMD boxes ensured standardized food uptake by all fasting participants.

Other studies examining lifestyle interventions showed similar results regarding sperm quality. For example, Montano et al. (31) conducted a RCT assessing the effect of a lifestyle intervention on the semen quality of healthy young men. The four-month program involving a mediterranean diet and moderate physical activity was shown to have a positive impact on semen quality, particularly on sperm concentration, sperm motility and the proportion of spermatozoa with normal morphology. Additionally, it resulted in a reduction in round cell concentration (31). These results suggest once more the high value of lifestyle interventions when it comes to subfertility. However, in that study a dropout rate of 28.1% was reported during the 16 weeks of the study, whereas in our study all fasting participants were able to comply with the three FMD cycles. Thus, an FMD-based intervention might be easier to conduct than a long-term dietary intervention.

Another dietary intervention study showed that men with obesity receiving a 800 kcal/day low-energy diet for 16 weeks profited more regarding semen quality than a control group with a brief dietary intervention. However, sperm quality also increased in the control group indicating that even mild dietary interventions could potentially improve semen quality (32). This is particularly interesting in the light of our study, as the 800 kcal intake diet can be compared to our FMD in some respects. A meta-analysis showed that bariatric surgery alone is not suitable to improve sperm quality (33). Thus, the improvements in semen quality repeatedly reported after weight loss might result from metabolic switches connected to caloric restriction rather than being attributed to the weight loss alone.

A recent study investigating the effects of Ramadan fasting on sperm quality reported that semen samples collected during the month-long fasting, showed decreased progressive motility and diminished semen volume compared with samples obtained from the same individuals outside of the Ramadan period (34). We hypothesize that semen quality could first decrease during periods of caloric restriction and improve during refeeding hormetically. If this hypothesis was confirmed it would underline the importance of considering doing research about the dynamics of sperm quality during and after caloric or dietary restrictions to time fertility treatments accordingly.

In our intervention group all parameters except ejaculation volume increased in the FG. As ejaculation volume is one parameter among other more meaningful parameters to assess fertility, such as sperm motility, concentration and total sperm count, it might be of less importance but should be monitored more closely in future studies. Concerning our qualitative findings, the desire to have children was a big motivator for participants to complete the intervention. Fasting gave them a feeling of self-efficacy concerning their desire for a child and encouraged positive lifestyle changes.

Fertility research to date mainly addresses women. In our interviews we were able to show that men also report important insights such as motivational reasons for lifestyle changes but also barriers in fertility treatment, which need to be considered to create a more equal approach for addressing infertility and subfertility in both genders.

Further, we agree with Kimmins et al. (4) who state the need for more research about male infertility worldwide given that the increase in methods for assisted reproduction (MAR) places the burden of infertility treatment inordinately on women, despite 50% of the cases affecting or at least involving the male partner. MAR are invasive procedures, which are being used increasingly worldwide, and are being progressively developed in their effectiveness. Parallel to this, less invasive and more individualized strategies could potentially support outcomes and should be investigated further. Research in men’s health is a crucial step to achieve gender equality in reproductive health care. Another aspect suggesting intensified research about subfertility is that understanding sperm quality as an expression for general men’s health might be crucial in becoming aware of potential co-morbidities and preventing them (4). Thinking further, if fasting was found to be effective in improving sperm quality, it might also be a tool of prevention for healthy men regarding the decline of sperm quality in western countries and aging parents. To our best knowledge, this is the first prospective clinical study of the effects of fasting on sperm quality, while nutritional and other lifestyle interventions have already shown potential in this field. We hope that our study can contribute to the development of specific dietary interventions in male fertility.

## Conclusion

In summary, our study aligns with other research findings, indicating that intensive dietary interventions could be linked to improved semen quality in men in some parameters, highlighting the need for further research concerning prevention and treatment of male subfertility.

## Data Availability

The raw data supporting the conclusions of this article will be made available by the authors, without undue reservation.
